# Electron spin resonance (ESR) dose measurement in bone of Hiroshima A-bomb victim

**DOI:** 10.1371/journal.pone.0192444

**Published:** 2018-02-06

**Authors:** Angela Kinoshita, Oswaldo Baffa, Sérgio Mascarenhas

**Affiliations:** 1 Departmento de Física, Faculdade de Filosofia, Ciências e Letras de Ribeirão Preto, Universidade de São Paulo, Ribeirão Preto, São Paulo, Brazil; 2 Pró Reitoria de Pesquisa e Pós-Graduação, Universidade Sagrado Coração, Bauru, São Paulo, Brazil; 3 Instituto de Física de São Carlos, Universidade de São Paulo, São Carlos, São Paulo, Brazil; Oklahoma State University Stillwater, UNITED STATES

## Abstract

Explosion of the bombs in Hiroshima and Nagasaki corresponds to the only historical moment when atomic bombs were used against civilians. This event triggered countless investigations into the effects and dosimetry of ionizing radiation. However, none of the investigations has used the victims’ bones as dosimeter. Here, we assess samples of bones obtained from fatal victims of the explosion by Electron Spin Resonance (ESR). In 1973, one of the authors of the present study (SM) traveled to Japan and conducted a preliminary experiment on the victims’ bone samples. The idea was to use the paramagnetism induced in bone after irradiation to measure the radiation dose. Technological advances involved in the construction of spectrometers, better knowledge of the paramagnetic center, and improvement in signal processing techniques have allowed us to resume the investigation. We obtained a reconstructed dose of 9.46 ± 3.4 Gy from the jawbone, which was compatible with the dose distribution in different locations as measured in non-biological materials such as wall bricks and roof tiles.

## Introduction

Electron Spin Resonance spectroscopy allows the use of constituents of the human body as dosimeter. It has been widely studied and explored due to its application in retrospective dosimetry in cases of accidental exposure to radiation. This is a subject of great relevance if we consider that advanced medical treatments, like the use of stem cells and transplantation, can be offered to the victims of such exposure depending on the radiation dose they received [1–3].

It is well established that ESR dosimetry of tooth enamel can be applied to assess radiation doses retrospectively. The International Atomic Energy Agency published a technical report–IAEA-TECDOC-1331 –that provided the guidelines for the application of tooth enamel as an ESR dosimeter. Tooth enamel is a very sensitive material for ESR dosimetry because its high density and high degree of crystallinity remain practically unaltered during life [4,5]. Since 1996, three laboratory inter-comparisons have been made to measure the accuracy of this dosimetry [6,7]. The third inter-comparison, finalized in 2006, demonstrated that ESR tooth dosimetry can detect doses as low as ~100 mGy [8]. Therefore, this technique is useful for the triage of victims, and it can help to define the best possible treatment for such victims [9,10].

Although the way of obtaining the dose is not straightforward, in specific cases, ESR dosimetry of bone is also a powerful technique for obtaining data on the received dose. One example is the case of systemic radiotherapy in which the radiopharmaceutical is directed to the bone region so, in this situation the ESR bone dosimetry is quite useful to evaluate the dose effectively deposited in bone [11–13].

The ESR spectrum of irradiated bone is composed of the radiation-induced signal, due mainly to the CO_2_^-^ [14] radical produced in the hydroxyapatite and another, broad signal, due to the other components. For doses of <4Gy, the interference of the broad signal can prevent dosimetry [15]. So, methods for isolating the dosimetric signal, in order to obtain a more precise dosimetry are discussed by Wieser [15] and Breen and Batista [16] that used physical and chemical methods. More recently, De[17] used higher frequency (Q Band) that presents better spectral resolution, allowing better visualization of the components and also the use of a smaller amount of material, with a mass comparable to the one obtained in a biopsy.

Another characteristic of bone dosimetry is the possible influence of bone remodeling that may, over time, destroy the signal induced by ionizing radiation. So, in a situation in which the analysis is performed after a period after irradiation, this factor must be taken into account and the value found by the ESR dosimetry will be the minimum value received by the victim. This is the case described by Desrosiers [18] that applied the ESR bone dosimetry to evaluate the dose received by two victims of an accident with a source of cobalt that led to the victim’s legs amputation. Fragments from femur, tibia, phalange were analyzed, showing the estimated distribution of doses received. The doses agreed to visual clinical exams of epidermal damage, but the author draws the attention to the influence of bone metabolism in the dose. This aspect of bone dosimetry was also observed by Kinoshita et al [19], in another case of accident with a cobalt source, in which the victim had his finger amputated four years after the accident. The comparison with the Fluorescence *in situ* Hybridization (FISH) dosimetry shows that the value of the dose received is greater than that directly found in the bone by ESR, due to bone remodeling. More recently Krefft [20] analyzed bones extracted from the mandible of radiotherapy patients who, for medical reasons, had to undergo the procedure. They found that 3 and 6 months after the irradiation the doses were consistent with those calculated by treatment planning systems. But in a bone removed 6 years after radiotherapy, the dose found was ∼14% lower than the calculated one. In these situations, bone dosimetry provides the minimum value of the dose received by the victim.

Another interesting aspect was pointed out by Degteva [21] and Shishkina [22]. When there is absorption of radioactive material that is incorporated into hard tissues as bone and tooth, as in the case of downstream settlements residents of Techa River, who were contaminated by ^90^Sr. The internal radiation rate of the radioisotope must be considered in the evaluation of the dose received externally.

Other papers involving accidents with radioactivity were reported bySchauer[23] and Clairand [24]. Schauer[23] reported an accident with a 3MV accelerator, and additionally, determined that for the same value of absorbed dose, the intensity of radiation induced ESR signal is different for a 0.6MeV electron source in comparison to 1.0 MeV, 2.5 MeV electrons, and ^60^Co gamma source. And that there is no difference in the intensity between the latter three. Clairand[24] determined the dose distribution in various organs of victims from very-high activity ^90^Sr sources accidents occurred in Georgia, using the ESR bone dosimetry results in association with Monte Carlo calculations, showing methodological advances and the utility of the technique in extrapolating other data of clinical relevance.

Explosion of the atomic bombs in Hiroshima and Nagasaki in 1945 was one of the most important radioactive events that occurred in the past. This event probably inspired the theme “retrospective dosimetry” and raised awareness of the need to evaluate radiation doses in accidental cases. The literature about this topic is vast and has consequences to the radiation protection of the population.

Shortly after the atomic bombing in 1945, many efforts started being made to assess the effects of ionizing radiation, which in turn depend on estimating the doses received by the victims. The works of Arakawa [25] and Higashimura [26], published in 60’s, and the work of Mascarenhas et al. [27] and Ikeya [28], published later, are worthy of note. Arakawa [25] reported data on the dose of radiation that victims and survivors received on the basis of what was known at the time. Arakawa performed his research in conjunction with Programs/Commissions created in Japan and the United States that were dedicated to studying the effects of the Atomic Bomb (US Atomic Energy Commission, Atomic Bomb Casualty Commission, Hiroshima-Nagasaki) at that time. Arakawa’s work described physical factors of the bombings such as the geometry of detonation in cities, the fission process, the possible products, the decrease in gamma and neutron radiation with distance from the hypocenter (based on previous work), and shielding history reported by survivors. The scattered radiation data were obtained by dosimetry of sites where weapons were tested. These data allowed estimation of the scattered radiation dose produced by a wall and buildings. To estimate shielding conditions better, Japan-type houses were built on the test site in Nevada to determine the attenuation of gamma and neutron radiation. The author emphasized issues related to the accurate determination of dosimetry.

In 1963, Higashimura et al. [26] published the first experimental results on the dosimetry of gamma radiation. These authors conducted TL experiments on roof tiles collected in the cities of Hiroshima and Nagasaki to detail how the dose was distributed as a function of the distance from the hypocenter; the authors then compared the experimental data with theoretically estimated results. Ichikawa et al. [29] later complemented this work.

As an attempt to use biological materials collected from the victims themselves, in August 1972 one of the coauthors of this paper (SM, Mascarenhas) visited Japan, contacted Japanese scientists working on nuclear medicine, and obtained human samples from the atomic bomb site. Professor Takeshita, a member of the Institute of Nuclear Medicine in Hiroshima, supported Mascarenhas’s ideas. Professor Takeshita had himself been in Nagasaki at the time the A-bomb exploded. In cooperation with Dr. Hasegawa of the University of Hiroshima, Professor Takeshita provided Mascarenhas with some valuable bone samples from different sites. A clear and strong signal indicating the magnetic memory effect due to radiation emitted by the A-bomb explosion was detected. By comparing the amount of magnetism induced with that produced when samples were exposed to Cobalt 60 gamma ray source, the researchers were able to estimate the radiation dose received by the samples in 1945. This sample was left in Japan. A preliminary account of the findings was presented at the American Physical Society Meeting held in April 23–26, 1973, in Washington, D.C. [27]. However, this estimate was not able to separate the radiation-induced signal from the background signal of the sample.

Similarly, Ikeya et al. [28] carried out ESR dosimetry on shell buttons and tooth enamel donated by survivors. In their paper, the authors mentioned the efforts made by Mascarenhas to measure radiation doses through bones, and they remembered that the attempt was primarily negative. Later, animal experimentation confirmed that radiation i*n vivo* produced paramagnetism in bones [30].

Sample bone from the collection mentioned above were brought to Brazil, where they have been stored since then. In this paper, the deposited dose in this bone sample from one individual exposed relatively close to the hypocenter of Hiroshima A-Bomb explosion was determined, demonstrating the potential of ESR bone dosimetry to the validation of Hiroshima dosimetry system using available biological samples.

## Materials and methods

### Samples

**Fig 1:**
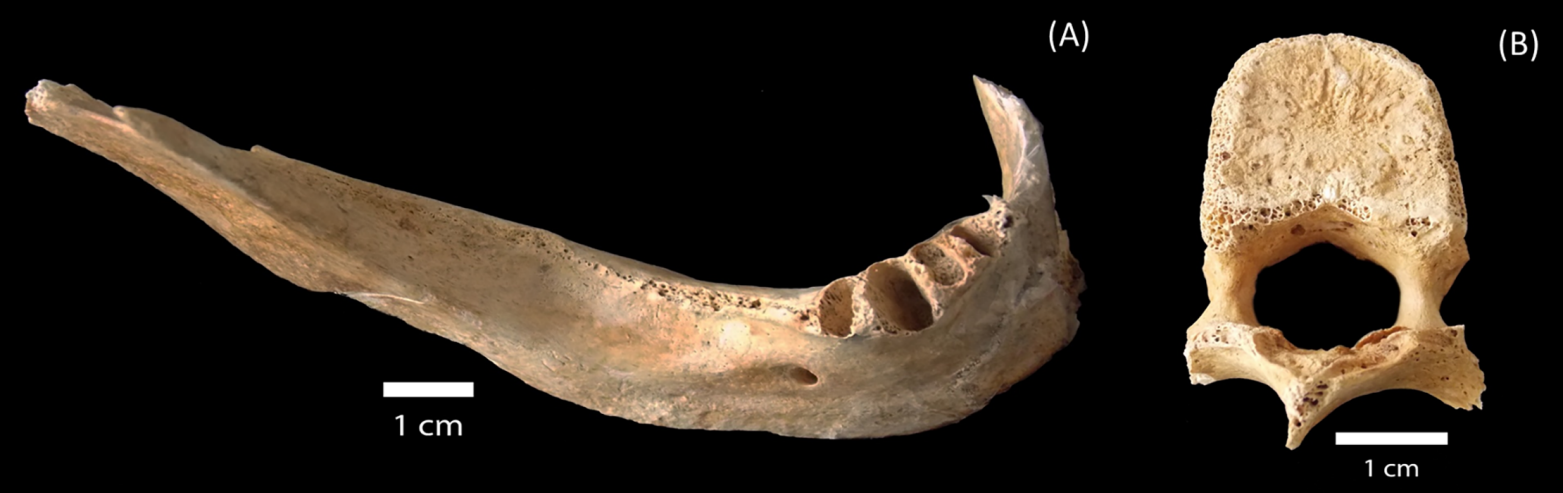
Victim’s mandible collected at Hirose and Nakahiro-machi.

A. Hasegawa and K. Takeshita, from the University of Hiroshima, provided Prof. S. Mascarenhas with the sample used in this work. Since then, the material has been deposited under his responsibility (Instituto de Física de São Carlos, Universidade de São Paulo). Fig 1 shows the part of a human mandible that was studied here in the way it was received, that is, clean and dry. This sample was stored in a light-tight box in the laboratory and was protected from direct sunlight and other possible agents that could induce radicals in the bone.

This mandible sample was collected at Hirose and Nakahiro-machi, which is located between 1250 and 1500 meters away from the atomic bomb hypocenter.

The mandible has an outer layer of cortical bone, compact and dense that was adequate for dosimetry studies.

### ESR dosimetry

A portion of the cortical bone of the region of the mandible body weighing approximately 2 g was carefully removed for the experiment. The sample was washed in water, oven-dried at 40°C, and used in the experiment. After drying, the material was carefully crushed in an agate mortar. Spectra of this sample were recorded in the X-Band Jeol Spectrometer to verify the presence of the radical induced by ionizing radiation. The measurement conditions were: Center Field = 345 mT, Sweep Width = 15 mT, Sweep time = 1 min, Modulation frequency = 100 kHz, Modulation Width = 0.1 mT, frequency = 9.4 GHz, and Microwave Power = 1 mW. A total of 400 scans were accumulated, which afforded a good S/N ratio. The g maker composed of Mn^2+^ in a MgO matrix was used to determine the g values of the signal precisely.

Simfonia (Bruker) software was used to analyze the signal. Initially, an isotropic signal with g = 2.0045, present in all bones [13,31,32], was subtracted from the experimental spectrum. This signal did not correspond to the deposited dose. Hence, it was a confounding signal that had to be removed from the spectrum. The resulting spectrum was then fitted to a simulated signal with spin Hamiltonian of axial CO_2_^-^, which has the following g values: g_⊥_ = 2.0025 and g_//_ = 1.9973 [14,33]. The agreement between the signals confirmed the presence of radicals induced by radiation from the atomic bomb. To determine the dose received by the sample, the additive dose method was applied.

The prepared sample was separated into 11 parts that weighed approximately 100 mg. Each part was irradiated with a different dose that ranged from 5 to 100 Gy, and the dose-response curve was constructed. The sample parts were irradiated in an animal irradiator device equipped with a 150-kV X-ray tube. A dosimetry of this unity is frequently accomplished to check the delivered doses. At the time of the irradiations, the dose rate was 1.27 ± 0.06 Gy/min in air [34].

Each irradiated fraction had its spectrum recorded under the same conditions described above. However, the signal was stronger, so a smaller number of spectra were accumulated. The intensity of the dosimetric signal, determined by simulation, was used to construct the dose-response curve. Extrapolation of the curve to Intensity = 0 allowed us to determine the dose received by the sample. Origin 8.5 software was used to draw the depicted plots.

## Results

**Fig 2:**
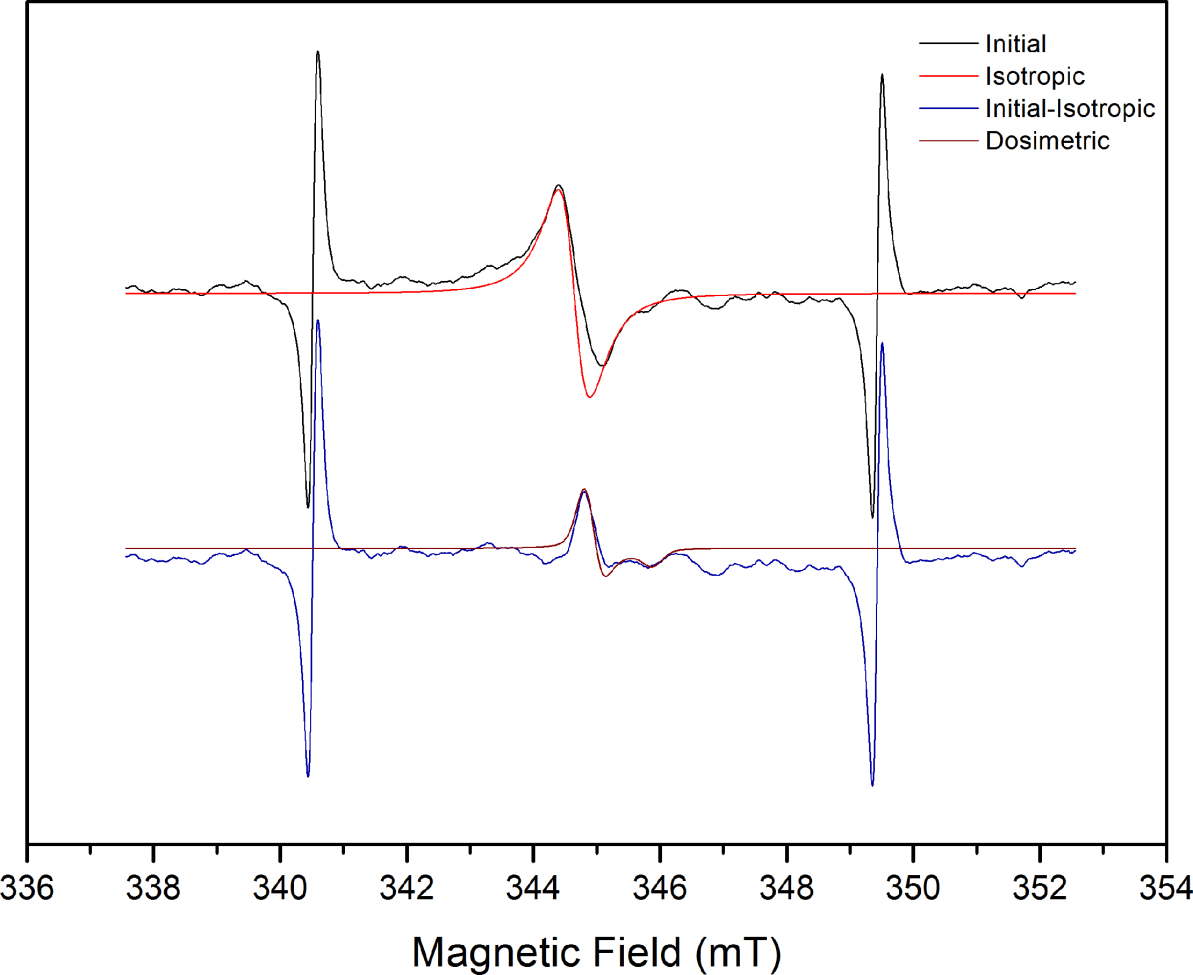
ESR spectrum of the mandible. Initial spectrum obtained after 400 scans of the mandible (black line) and simulated isotropic radical at g = 2.0045 (red line). Fitting of the signal after subtraction (blue line) with the axial CO_2_^-^ signal Hamiltonian parameters (g_⊥_ = 2.0025 and g_//_ = 1.9973). The two high-intensity sidelines are the Mn 3^rd^ and 4^th^ lines used as marker to determine the g values precisely.

Fig 2 shows the initial spectrum obtained after 400 scans of the mandible (black line) and the spectrum of the simulated isotropic radical at g = 2.0045 (red line). The signal obtained after subtraction (blue line) fitted the signal of axial CO_2_^-^, recognized as a radical induced by incidence of ionizing radiation on hydroxyapatite (Hap). After irradiation, the signal intensified. Therefore, there was no saturation, as seen from the increasing signal-to-noise ratio with increasing deposited dose.

**Fig 3:**
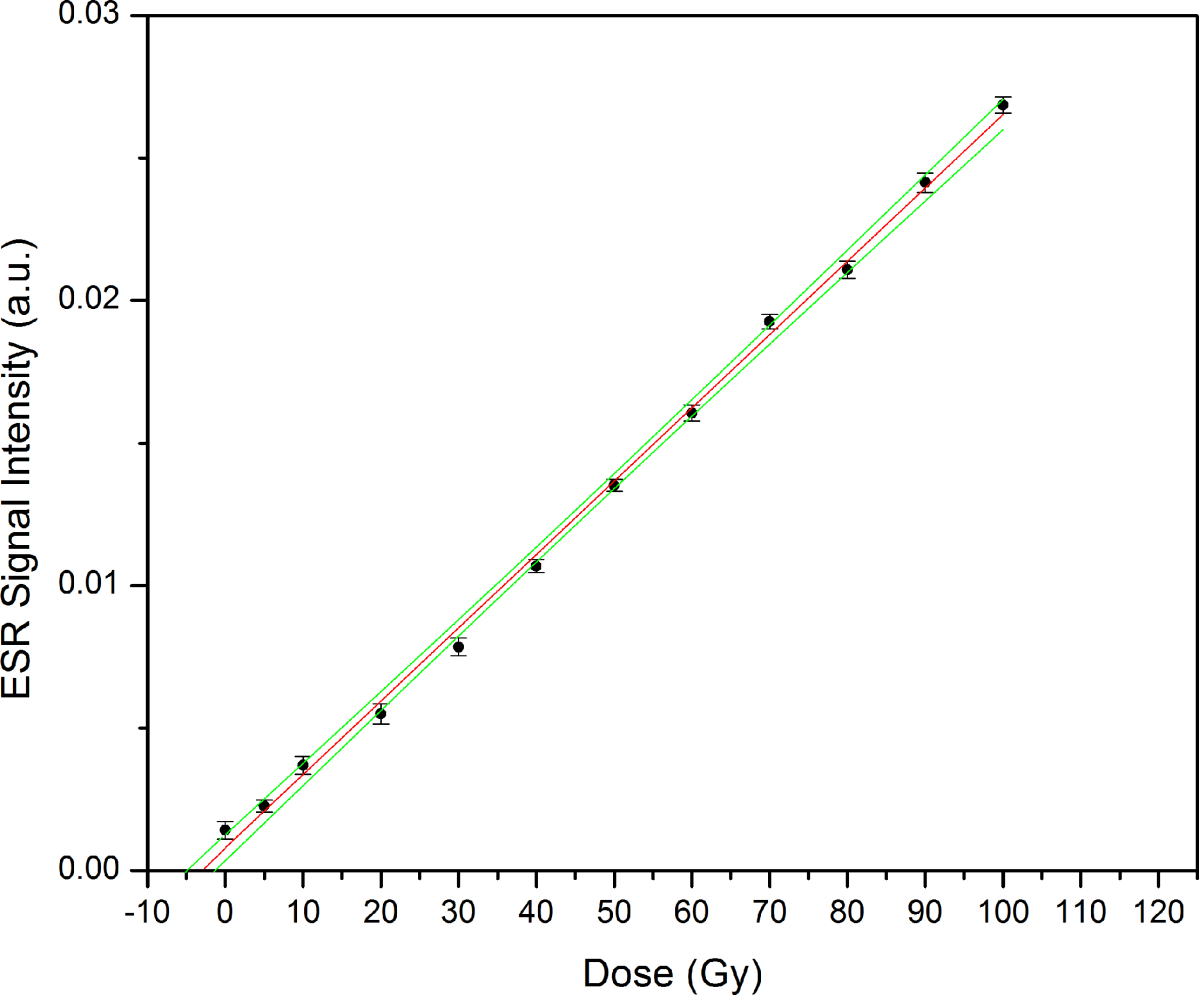
Experimental data and dose-response curve obtained by linear fitting. The received dose was 3.10 ± 0.84 Gy. The green lines show the 95% confidence band interval, 95% UCL, and 95% LCL.

Fig 3 illustrates the dose-response curve. As recommended, at least 10 experimental points spanning 10 times the expected dose must used [35]. The fitting of experimental data in ESR dosimetry can sometimes be a matter of debate, but in our case we employed a linear fitting to adjust the experimental data points because the coefficient of determination (r^2^) was 0.9977. We obtained the following equation:

$$I=I_{0}(1+\frac{D}{D_{E}})$$ (1)

with a Person´s r factor of 0.999 and I_0_ of 7.97.10^−4^. The value of D_E_ was 3.10 ± 0.84 Gy for the reconstructed dose. This value corresponds to dosimetry of the source used, that is the dose of 150-kV X-ray tube in air.

For comparison with other data from literature, another aliquot was irradiated with 50Gy (dose in water) using a linear accelerator LINAC (Mevatron 12 Siemens 6 MV). After extraction of dosimetric signal, the intensities were compared and a factor of 3.3 ± 0.3 was obtained. So, after conversion of the result due to the different radiation energy employed, the dose is 10.2 ± 3.7 Gy (dose in water). Using the μ_en_/ρ provided by NIST [36] this dose corresponds to 9.46 ± 3.4 Gy (in bone) and 9.16 Gy± 3.3 Gy (in air).

## Discussion

ESR dosimetry in bones is not novel. Many papers have dealt with reconstruction of the radiation dose received during radiological accidents. However, the samples analyzed in this work have important historical value because they belong to fatal victims of the first and only moment in history when nuclear weapons were used against civilian targets. This work demonstrates how technological advances, such as higher sensitivity of spectrometers and use of software to simulate ESR spectra, can contribute to the conduction and finalization of investigations that did not advance to final conclusions in the past.

Investigators accomplishing retrospective dosimetry face numerous ill-disposed difficulties, like preservation of the sample and dependence of the ESR signal on radiation energy, dose rate, and temperature of irradiation, among other factors. The sample used in this work was well preserved and had no signs of overheating or burning. The location of the sample led us to assume that the radiation field consisted mainly of scattered high-energy photons and gamma radiation. One complicating factor was the dose rate: ideally, the signal versus dose plot constructed in the laboratory should be obtained in conditions where the signal emerging at a certain additive dose was the same as the actual signal the sample would have presented at the site of the event, even though the dose rate was very different. In archeological dating, samples in the natural environment are irradiated at a very low dose rate, typically mGy/year. Nevertheless, samples irradiated in the laboratory receive dose rates of 10–100 Gy/year, which is 10^8^ higher than the dose they receive in the natural environment. Hap, the mineral part of hard tissues, has been extensively used for dating. Hap provides archeological or equivalent doses that are consistent with the age of the materials. In the case of the explosion of the A-Bomb, the event lasted about 0.1 μs. Using the dose measured in this work the sample was exposed to a dose rate of about 10^7^ Gy/s that is about 10^7^ higher than the dose rate used to construct the signal versus dose, which was the same order of magnitude as the dose rate used in dating works, but it in the opposite direction. Therefore, even if we used dose rates that were much smaller than the actual doses at the site of the bombing event, the total dose found herein could be trusted.

The results obtained here agreed with the data reported by Higashimura [26], who found doses ranging from 7 to 9 Gy at sites located 970 m away from the hypocenter, Although the author does not identify the medium in which the dosimetry was based, as the values are reported is in rad, we suppose that it refers to dose in the water. Our results were also in line with data described by Ichikawa [29], who detected doses varying from 370 to 600 R at sites located 980 m away from the hypocenter. The unity R (of exposure) probably refer to dose in air. Differences between our results and literature data lay on the fact that the bones analyzed herein were located a little further from the hypocenter. Another difference was the scattering and attenuation effects of the building materials of the samples used here (Hap) and in references [22] and [25] (quartz). Our results also agreed with data published by Ikeya [28], who found (2.1 ± 0.2) Gy in the shell button and (3.1 ± 0.5) Gy in dental enamel. These values were compared with the dose estimate report and were discussed for shielding factors T65D [37].

Nakamura [38] carried out an extensive study that correlated ESR dosimetry in the teeth of surviving victims with cytogenetic dosimetry. Although almost 300 teeth were donated to this author, he was not able to use all of them. Analysis of blood samples from 61 of the 69 surviving donors of the teeth, who resided within 2 km of the hypocenter, and of the DS86 dose estimate data [39] available in the literature revealed differences in the dose received by the lingual and buccal region of the teeth and demonstrated that the dose depended on the region the teeth was located in the oral cavity, molar or frontal. The average dose on the buccal part of the front teeth (1.85 ± 0.73 Gy) was significantly higher than the average dose on the buccal part of the molar teeth (1.07 ± 0.55 Gy). The average dose on the lingual part of the front teeth (1.35 ± 0.67 Gy) was higher than the average dose on the lingual part of the molar teeth (1.06 ± 0. 52 Gy). The author concluded that dosimetry on the buccal part of the front teeth could overestimate the dose. They found a close relationship between ESR dosimetry results for the lingual portion of the molar teeth and the cytogenetic biodosimetry based on translocations. The doses reported refer to doses in air.

More recently, Imanaka [40] published a work that precisely reviewed all the history of the studies and of the commissions that investigated various aspects related to radiation dosimetry in Hiroshima and Nagasaki. The work reported substitution of the dosimetric system DS86 for the dosimetric system DS02 [40].

In summary, the present work shows that it is essential to treat ESR signals from bones, which could be a useful material for retrospective dosimetry in the case of Hiroshima. This approach could open new possibilities to measure doses at the bombing site with available material and correlate to the theoretical dosimetry system.
